# Surface temperature elevated by chronic and intermittent stress

**DOI:** 10.1016/j.physbeh.2018.04.004

**Published:** 2018-07-01

**Authors:** Katherine A. Herborn, Paul Jerem, Ruedi G. Nager, Dorothy E.F. McKeegan, Dominic J. McCafferty

**Affiliations:** a Institute of Biodiversity, Animal Health & Comparative Medicine, University of Glasgow, Jarrett Building, Glasgow G61 1QH, UK; b Institute of Biodiversity, Animal Health & Comparative Medicine, University of Glasgow, Graham Kerr Building, Glasgow G12 8QQ, UK; c Institute of Biodiversity, Animal Health & Comparative Medicine, University of Glasgow, Scottish Centre for Ecology and the Natural Environment, Rowardennan, Drymen G63 0AW, UK

**Keywords:** Body temperature, chronic stress, enrichment, welfare, *Gallus gallus domesticus*, infrared thermography

## Abstract

Stress in homeothermic animals is associated with raised body core temperature and altered patterns of peripheral blood flow. During acute stress, peripheral vasoconstriction causes a short-lived drop in surface temperature that can be detected non-invasively using infrared thermography (IRT). Whether and how skin temperature changes under chronic stress, and hence the potential of IRT in chronic stress detection, is unknown. We explored the impact of withdrawing environmental enrichments and intermittent routine handling on long-term skin temperature in laying hens (*Gallus gallus domesticus*). Immediately following enrichment withdrawal, comb, face and eye temperature dropped, suggesting this was acutely stressful. In the 3 weeks that followed, barren-housed hens displayed behavioural markers of frustration. Whilst control birds, housed in enriched conditions, showed a decline over weeks in both comb temperature and baseline corticosterone levels, barren-housed hens had no change in comb temperature and an increase in corticosterone. By the trial end, comb temperature (but not corticosterone) was significantly higher in barren-housed hens. This change in parameters over time may reflect cumulative impacts of enrichment withdrawal in barren pens and/or, as hens were young and maturing, age-related changes in controls. Comb, face and eye temperature were also higher on days following routine handling, and comb temperature higher on other days in hens that were regularly handled for blood sampling than for a less intensive weighing protocol. Together, these data support comb, face and eye surface temperature increase as a long-term marker of stress exposure in laying hens. It is important to recognise that the strength and even direction of these effects may vary with thermoregulatory and energetic context. However, in laboratory and indoor-reared farm animals that live in carefully managed environments, IRT of the skin can potentially be used to non-invasively monitor chronic and intermittent stress exposure.

## Highlights

• We measured surface temperature (ST) profile in hens subject to long term and intermittent stress.
• The impact of stressors was validated using established behavioural and hormonal markers.
• Enrichment withdrawal caused a short term drop in ST but living in a barren environment increased ST.
• Hens also had higher ST on days following handling or when subject to more intense handling methods.
• Thermal imaging of ST offers a non-invasive approach toward chronic stress monitoring.

## 1 Introduction

The possibility that animals experience positive and negative emotions places the measurement and minimisation of stress at the heart of animal welfare [32]. Stress causes an increase in core body temperature [36]. When acute, this is transient: peripheral vasoconstriction shunts blood into the core, which increases core body temperature, decreases surface temperature, and also channels nutrient supply to the organs to support metabolism, hence heat production [37]. On the body surface, this is evident as a short-lived pattern of skin cooling (vasoconstriction), warming (vasodilation for core heat dissipation) and then recovery [31]. The magnitude of this response differs between stressors of different intensity; thus, surface temperature may be used not only to identify but to quantify acute stress in animal welfare assessment [19]. Chronic stress is arguably a greater concern in the welfare of domestic animals, however. In rodents, core temperature increased with chronic stressors such as small or barren cages [28] or recurrent interactions with distressed [15] or dominant conspecifics [39]. As such, core temperature is supported as a welfare indicator for chronic stress. If chronic stress increases core temperature, it may also be characterised by processes that either conserve or dissipate core heat, with opposing impacts on surface temperature. Whether and how surface temperature changes with chronic stress is little researched [23] and therefore harder to predict.

Humans experiencing long term psychological stress, with chronic fatigue syndrome, have a relatively high skin temperature [40] and prefer to bathe their hands in cool rather than warm water [36]. These are autonomic and behavioural responses to dissipate core heat, thus we may predict elevated surface temperature as a marker of chronic stress. Supporting this prediction, Foster and Ijichi [16] report a positive correlation between eye temperature, measured with infrared thermography (IRT), and stress-responsive personality score in shelter cats (*Felis catus*), which may reflect higher hormonal stress-responsiveness. However, a study on wild blue tits (*Cyanistes caeruleus*) observed the opposite: high baseline corticosterone levels, suggestive of chronic stress state, correlated with *low* eye canthus temperature [23], possibly due to longer-term peripheral vasoconstriction to conserve heat. In this case, eye temperature was in addition lower in birds with poor body condition scores, and lower on colder days. In attempting to isolate stress effects, this result highlights the importance of considering also the well-established roles of peripheral vasoconstriction and vasodilation in the management of heat loss for energy conservation and thermoregulation, where extremes of resource availability or temperature may indeed be a source of chronic stress. Thus, context may alter the intensity or potentially direction of stress-induced surface:core temperature relationships [29,33]. In indoor-housed farm and laboratory animals, though, feed intake and climate, hence potential energetic and thermoregulatory influences on body temperature, are carefully managed. As such, identifying consistent, directional changes in surface temperature with chronic stress from a context-specific norm could be of great applied value to welfare assessment in these animals.

We explored the impact of two stressors - barren housing and intermittent handling - on comb, face and eye surface temperature in laying hens (*Gallus gallus domesticus*) housed at 18 °C (commercial management recommendation; [46]) with *ad lib* food availability. We used IRT to measure surface temperature: an approach already proposed for non-invasive assessment of acute welfare concerns such as husbandry interventions [45]. Pre-laying and nest-building behaviour, perching at night and foraging opportunity are defined as behavioural needs in laying hens [48], i.e. psychological suffering may be incurred where they cannot be expressed [21]. Previous studies indicated that laying hens prefer and will work to access environments with perches [20,38] and nest boxes [7,9]. Provisioning with straw and foraging enrichments appears to reduce frustration, lowering rates of feather pecking [35] and stereotypic object pecking, respectively [10]. Thus, our chronic stress treatment was to withdraw perches, nest boxes, straw and foraging enrichments. These enrichments were visible but inaccessible rather than completely withdrawn, which may maintain chickens in a long-term state of frustration [38]. Handling is an acute stressor in poultry, where increasingly restrictive holds (e.g. cradling upright versus pinning on the side) are increasingly stressful, inducing greater magnitudes of hormonal and comb temperature responses [19]. The impact of such acute stressors is mediated by the broader stress context [24], for example in Japanese Quail (*Coturnix coturnix japonica)*, the negative, stress-induced impacts of handling on immune function were worse in birds housed in barren than enriched pens (Nazar & Marin, 2010). Therefore, enrichment may serve not only to improve baseline welfare but also coping under acute stress.

In the experiment, we examined the impacts of 23 days of enrichment withdrawal combined with weekly handling at two intensities (cradling for weighing and side-pinning for blood sampling) on the comb, face and eye surface temperature of laying hens using IRT. We cross-validated the effect of our manipulation using established hormonal and behavioural markers. Our objective was to determine whether enrichment withdrawal and handling triggered a consistent direction of change in skin temperature, and hence the potential of IRT as a tool for the non-invasive measurement of chronic stress in free ranging animals.

## 2 Methods

### 2.1 Subjects and husbandry

Data were collected from July–September 2014 at Cochno Farm & Research Centre, University of Glasgow. Fifty-six 16-week old Lohmann Brown pullets were obtained from a commercial supplier. On arrival, they were fitted with an identifying leg ring and randomly allocated to 14 pens across two rooms in groups of 4. Each pen was one half of a larger, opaque-sided 4.5m^2^ square pen, divided diagonally with mesh to allow visual contact between two adjacent, triangular “pen pairs”. A litter of wood shavings was replaced weekly. Commercial layers feed and water were provided *ad libitum*. The rooms were maintained on a 14 h:10 h light:dark cycle at 18 °C and 50–60% relative humidity (mean ± s.d. in two rooms during testing: 18.29 ± 0.16 °C and 52.6 ± 2.5%, 18.29 ± 0.21 °C and 53.7 ± 3.1%).

**Fig. 1:**
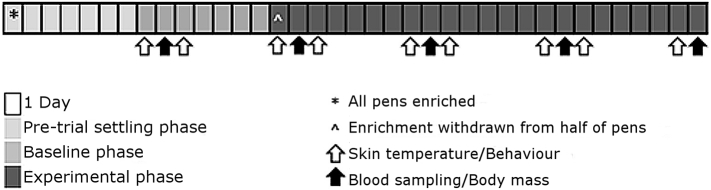
Schematic of the trial

All hens were confirmed to be laying within 3 weeks of arrival by checking until 4 eggs were routinely collected from each pen per day. Enrichments were then provided to all pens: a 1.5 m dowel perch, nest box (40 cm x 30 cm x 30 cm; roof of which also used as a perch), straw, daily scattering of 50 ml of mixed corn and weekly scattering of 50 ml grit into the litter and, on Mondays (after filming, see below) and Fridays, a halved cabbage, which was well pecked but largely uneaten on replacement. Hen pullets are expected to adapt their behaviour within a week of changes in the husbandry environment [8], thus were allowed a one week settling phase in this enriched condition before data collection began (see Fig. 1). The baseline phase entailed one week of surface temperature, behaviour and corticosterone data collection with all hens in the enriched condition. The experimental phase then began with the replacement of litter in all pens, so that all were disturbed, and at the same time removal of enrichments from half of the pens. Enrichments were removed from one of each pen pair, randomised to the right or left pen, so that they would be visible (in the neighbouring pen) but inaccessible to the hens in the barren pens. Data were collected in this “experimental phase” for 23 days. Body mass and, in a specific subset of hens, blood samples were collected on Tuesdays. To capture effects of this routine handling, thermal and behavioural data were collected on Mondays, when hens were 6 days from last handling experience thus relatively undisturbed, and on Wednesdays, henceforth “Undisturbed” and “Post-handling” days. Apart from on the day of enrichment withdrawal, litter was replaced in all pens on Thursdays.

### 2.2 Thermal video data

Thermal video was collected using a FLIR SC640™ camera (15 FPS, sensitivity 0.1 °C, accuracy ± 2%). On Undisturbed and Post-handling days, pens were filmed for a 2–3-h period between 10:50 and 13:45, except on the day of enrichment withdrawal when they were filmed between 13:20 and 15:20, 2–2.5 h after enrichment withdrawal. Each pen was videoed in a pre-determined random order until 1 min of unobstructed footage had been obtained for each hen in turn, with the order of hens also randomised. In both cases, all possible orders were listed and a different order drawn at random per day. Two (of 486) hen videos were excluded due to uncertainty over focal hen identity. When the focal hen was observed preening with closed eyes, filming did not start in the next 30 s to avoid error from warming of the eye by the eyelid [43]. Observations of preening were not collected sufficiently systematically to explore this as a marker of comfort [13], but did not appear to differ with treatment (27 control and 33 barren pen observations).

From each 1-min footage, still images of the focal hen with head side-on to the camera were selected at 10 ± 3 s intervals using the software FLIR ResearchIR™, with missing data points where no focussed image of the face was available. This resulted in a mean of 6 selected images per min (range 4–7, max. possible: 7). From the selected images, following Herborn et al. [19] we used a free-hand drawing tool in FLIR Thermacam Researcher Pro 2.10™ to draw around the comb and face (head excluding comb and wattle) to extract their maximum temperatures, and recorded the temperature at the centre of the eye. We have obtained >95% within-observer repeatability in demarcation of these facial regions from thermal images [19]. Unlike our previous study [19], we do not explore wattle temperature as here, water was available during videoing, thus skin cooling may have occurred through unobserved contact with drinker water prior to videoing. Emissivity was set to 0.97, and air temperature during filming (18.3 ± 0.4 °C) was included in analyses. In addition, for each image, we noted the following descriptors of body position that affect temperature estimation [19]: head position (above or below shoulders), head angle (facing ahead or toward the ground), head tilt (side on, or angled up to approximately 30° toward or away from the camera), side (right or left), and distance to the camera (ordinal categories: pen divided from front to rear into four equal parts). At the start and end of the trial, hens were photographed from the side and pixel count then converted to comb size relative to a scale in the image, using ImageJ™. Comb area did not significantly change within individuals during the trial period (Paired T test *t* = −1.81, p-value = 0.08) and start and end measurements were highly correlated (Pearson's correlation *r* = 0.94, *t* = 18.92, *p* < 0.0001), thus only one, initial comb area, was used as a covariate in analyses as a measure of comb surface area available for heat dissipation (see 2.4).

From the same thermal videos, behaviour could also be identified at the same 10 s intervals during the 1-min observation and was categorized as “active”: yes, no; “food-oriented”: foraging in the litter, using hoppers, no; “cage-pecking” (running bill along and pecking at caging): yes, no; and (“looking” (focal bird's body was oriented towards and within 2 body lengths of the neighbouring pen that had the opposite treatment): yes, no). Inactivity, reduced foraging, cage pecking and looking all may reflect disinterest in or frustration with the environment [10,30].

### 2.3 Corticosterone and body mass

A subset of twenty-eight hens, two per pen, was selected at random (drawn blind from a pool) at the start of the trial to examine treatment effects on baseline plasma corticosterone concentrations. Each Tuesday, these same hens were blood sampled and weighed whilst the remaining 28 hens were weighed only. As corticosterone shows a daily circadian rhythm in chickens [11], the order of pen and treatment sampling was randomised throughout the trial to avoid systematic biases. Repeatedly entering rooms to sample different pens may disturb, and hence elevate corticosterone, in subsequently sampled pens (peak corticosterone observed ~15 min post-acute stressor; [6]). To minimise potential disturbance effects, pens were arranged in rooms to preclude visual contact between all but pen pairs, so that capture was not generally overlooked by other hens. We allowed at least 30 min between entries to the same room and a longer recovery time of at least 1.5 h before returning for the second of pen pairs. Time of sampling (“Clock time”), a measure of the number of disturbances prior to sampling, was included as a covariate in the analyses (see 2.4). The two blood sampled hens for a focal pen were caught at the same time by two observers, carried in a box to an adjacent procedures room, sampled by syringe from the brachial vein within 3 min of entry to the home pen (mean 106 s from entry into the pen to sample completion, range 49–179 s) and then weighed. Plasma was separated by centrifugation at 5000 rpm for 2 min and frozen at −20 °C until analysis. On the blood-sampled hens' return, the two remaining hens were caught and weighed in the procedures room and returned. The categories “weighed only” versus “blood sampled” may also be interpreted as a handling stressor of a relatively low and higher intensity, respectively [19], so we controlled for Hold in the analyses of surface temperatures and behaviour (see 2.4).

Corticosterone concentrations were determined from 50 μl of plasma in 2 replicates. Following a standard, diethyl ether extraction (plasma vortexed with 5 ml diethyl ether, centrifuged, then solvent decanted using a methanol dry ice bath, dried and reconstituted in 600 μl ELISA calibrator diluent), a commercial ELISA kit (Caymen Chemical Company, Ann Arbor, USA) was used according to the manufacturer's instructions. Of 140 blood samples (5 per hen), 1 was too small to analyse, 1 was excluded due to inconsistency across sample replicates (coefficient of variation 0.20, mean c.v. ± s.d. = 0.043 ± 0.036), 2 due to sampling outside of 3 min, and 1 due to the hen laying an egg immediately after sampling.

### 2.4 Statistical analyses

Data were analysed in R version 3.3.3 (R Core Team, 2017, http://www.R-project.org/) with the packages nlme for Gaussian surface temperature and corticosterone data [41] and LME4 for binary behavioural data [3]. To account for repeated measures of the same individuals, we used Linear or Generalised Linear Mixed Models (LMMs or GLMMs) with hen ID as a random effect. All main effects were retained in our final models, but we performed a backwards stepwise elimination to determine whether also to retain specified interactions based on a significant likelihood ratio test (LRT) between consecutive models.

First, we examined the short-term effects of enrichment withdrawal on surface temperature (2–2.5 h post-withdrawal). We included the interaction of Treatment × Phase (categorical: baseline v experimental) to test for differences between treatments following manipulation. Covariates were Clock Time (expressed as min from midnight), Body Mass (g), Comb Size (pixel area relative to a scale) and Air Temperature (°C), which may all affect body temperature, and Distance to the camera (4 quarters of the pen expressed as continuous), four behavioural categories (Active, Cage Pecking, Food-Oriented, Looking) and four descriptors of head position (Head Angle, Head Tilt, Head Position, Side) to account for experimental noise influencing temperature measurement.

Second, we examined short-term effects of enrichment withdrawal on behaviour. The binary variables: Active, Cage Pecking, Foraging (versus other behaviours including using hoppers) and Looking were response variables in separate models, with a binomial error structure. As with the short-term surface temperature models, we included the Treatment x Phase interaction. Covariates were Clock Time, Body Mass and Air Temperature.

Third, we examined long-term changes in surface temperature during the 23 day experimental phase. The number of days of exposure to the manipulation was treated as a continuous variable, ‘Days of Manipulation’ (0 for baseline phase measurements), and we included the interaction of Treatment x Days of Manipulation to identify gradual changes in skin temperature in barren versus control hens. Day Type (Undisturbed versus Post-handling), Hold (Blood sampled or Weighed) and the interactions Treatment x Day Type, Treatment x Hold and Day Type x Hold were also included to explore how the impacts of intermittent handling were modified by enrichment, with time and with handling intensity. Covariates were otherwise as short term surface temperature analyses.

Fourth, we examined long-term changes in behaviour during the experimental phase. Models were formulated as the short-term behavioural analyses but including those additional interactions specified for long term surface temperature models. Logistic models did not converge with all four interactions however, thus with *a priori* predictions for the other three interactions, we omitted the Treatment x Hold interaction in these models.

Finally, we examined long-term changes in baseline corticosterone levels during the experimental phase. Corticosterone concentration (ng/ml) was log transformed to meet model assumptions. We again include the Treatment x Days of Manipulation interaction, but the terms Day Type and Hold and their interactions were not applicable as Corticosterone data was necessarily limited to handling days and blood-sampled hens. Clock Time, Body Mass, Air Temperature and Sampling Latency (seconds from entering the pen until sample collection) were covariates.

### 2.5 Ethical statement

Research was subject to review by the Animal Welfare and Ethical Review Board at the University of Glasgow and conducted under Home Office authority (Project license 60/4466). Hens were attended by an on-call veterinarian. No feather loss or significant injuries occurred. After trials, hens were rehomed by local hobbyists.

## 3 Results

### 3.1 Short term impacts of enrichment withdrawal on surface temperature

**Fig. 2:**
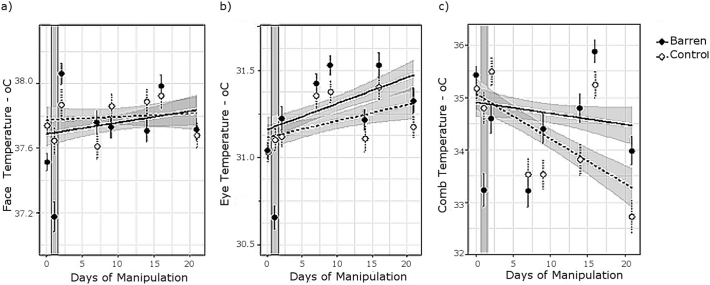
Change through the trial in a) Face, b) Eye and c) Comb temperature within treatment groups. Data at 0 on the x axis corresponds to baseline data. Confidence intervals are shaded around regression lines. Due to the large number of data points, the mean ± 1 S.E. per sampling day are instead shown for clarity. The shaded column indicates the skin temperature measurements collected in the 2–2.5 h immediately following enrichment withdrawal (not included in long-term analyses), to illustrate the short-term drop in temperature from baseline measurements in the barren group.

In all facial regions, there was a significant Treatment x Phase interaction: comb, eye and face temperature were all lowered at 2–2.5 h of enrichment withdrawal in barren pens, whilst surface temperature in control pens was unchanged from baseline (Table 1, Fig 2). Air Temperature correlated negatively with face and eye but not comb temperature (Table 1). The impacts of variables describing experimental noise are summarised in Table 1.

**Table 1:** Immediate effects (within 2–2.5 h) of enrichment withdrawal on 991 comb temperature, 991 face temperature and 979 eye temperature measurements from 56 hens. Key explanatory variables are underlined; other variables control for behaviour and body position relative to the camera at measurement. Data were analysed using LMM with hen identity as a random effect.

Variable	Comb			Face			Eye		
	Coef ± S.E.	*t*	*P*	Coef ± S.E.	*t*	*P*	Coef ± S.E.	*t*	*P*
Intercept	14.80 ± 21.20	0.70	0.49	59.9 ± 4.85	12.35	<0.0001	52.51 ± 5.16	10.17	<0.0001
Treatment - Control	−0.38 ± 0.54	−0.70	0.49	0.03 ± 0.08	0.36	0.72	−0.08 ± 0.10	−0.79	0.43
Phase - post-withdrawal	−3.48 ± 0.52	−6.64	<0.0001	−0.70 ± 0.12	−5.80	<0.0001	−0.80 ± 0.13	−6.21	<0.0001
Distance to camera	−0.67 ± 0.10	−6.86	<0.0001	−0.61 ± 0.02	−26.84	<0.0001	−0.25 ± 0.02	−10.52	<0.0001
Active - Yes	−0.42 ± 0.26	−1.65	0.10	−0.11 ± 0.06	−1.85	0.065	−0.30 ± 0.06	−4.79	<0.0001
Cage pecking - Yes	−0.04 ± 0.39	−0.10	0.92	−0.05 ± 0.09	−0.59	0.56	0.19 ± 0.10	1.99	0.047
Food-oriented – Hoppers	0.06 ± 0.33	0.18	0.85	0.24 ± 0.08	3.18	0.0015	0.93 ± 0.08	11.61	<0.0001
Food-oriented – Litter	−0.41 ± 0.34	−1.21	0.23	−0.14 ± 0.08	−1.81	0.071	0.46 ± 0.08	5.58	<0.0001
Looking – Yes	−0.42 ± 0.23	−1.87	0.062	−0.17 ± 0.05	−3.22	0.001	−0.04 ± 0.06	−0.74	0.46
Head angle – Down	0.44 ± 0.29	1.53	0.13	−0.12 ± 0.07	−1.82	0.069	0.08 ± 0.07	1.12	0.26
Head position – Up	−0.07 ± 0.38	−0.19	0.85	−0.14 ± 0.09	−1.52	0.13	−0.18 ± 0.09	−1.94	0.053
Head tilt - Side on	−0.69 ± 0.32	−2.14	0.032	0.49 ± 0.08	6.54	<0.0001	0.04 ± 0.08	0.45	0.66
Head tilt – Away	−0.46 ± 0.65	−0.72	0.47	0.30 ± 0.15	1.96	0.050	−0.07 ± 0.16	−0.43	0.66
Side – Right	−0.34 ± 0.19	−1.79	0.073	−0.04 ± 0.04	−0.80	0.43	−0.14 ± 0.05	−2.91	0.0037
Clock Time (min from midnight)	0.01 ± 0.00	2.07	0.038	0.00 ± 0.00	4.91	<0.0001	0.00 ± 0.00	5.00	<0.0001
Body mass (g)	3.36 ± 2.52	1.34	0.18	−0.22 ± 0.40	−0.54	0.59	0.32 ± 0.46	0.68	0.50
Air Temperature (°C)	0.68 ± 1.21	0.56	0.58	−1.33 ± 0.28	−4.77	<0.0001	−1.35 ± 0.30	−4.55	<0.0001
Comb size	0.00 ± 0.00	0.26	0.80	−0.00 ± 0.00	−0.07	0.94	0.00 ± 0.00	0.05	0.96
Phase x Treatment	2.27 ± 0.39	5.81	<0.0001	0.41 ± 0.09	4.48	<0.0001	0.44 ± 0.10	4.49	<0.0001

### 3.2 Short term impacts of enrichment withdrawal on behaviour

An increase in foraging with enrichment withdrawal was greater in control than barren pens, indicated by a significant Treatment x Phase interaction. The other behaviours: Active, Looking and Cage Pecking, did not differ between Treatments (Table 2a). Air Temperature correlated negatively with Cage Pecking and Looking (Table 2a). Other covariates were non-significant (Table 2a).

**Table 2:** Correlates of four binary behavioural categories scored in 56 hens. a) Immediate effects of enrichment withdrawal using 1162 point observations. b) Long-term effects of enrichment withdrawal using 3136 point observations. Data analysed using a GLMM with hen identity as a random effect and a binomial error structure. Test statistics in bold refer to interactions removed from the model based on a non-significant Likelihood Ratio Chi-squared test.

Fixed effect	Active			Cage pecking			Foraging			Looking		
a. Immediate impacts	Coef. ± S.E.	z/∑2	P	Coef. ± S.E.	z/∑2	P	Coef. ± S.E.	z/∑2	P	Coef. ± S.E.	z/∑2	P
Intercept	−12.53 ± 15.22	−0.82	0.41	18.51 ± 28.56	0.65	0.52	−67.59 ± 19.20	−3.52	0.0004	−0.16 ± 0.18	−0.88	0.38
Treatment – Control	−0.10 ± 0.25	−0.41	0.68	−0.56 ± 0.41	−1.39	0.17	−0.70 ± 0.39	−1.81	0.071	−0.20 ± 0.27	−0.76	0.45
Phase – Experimental	0.35 ± 0.38	0.97	0.36	−1.05 ± 0.70	−1.50	0.13	2.89 ± 0.51	5.63	<0.0001	0.37 ± 0.39	0.94	0.35
Clock Time (min from midnight)	−0.00 ± 0.00	−1.60	0.11	0.01 ± 0.01	1.64	0.10	−0.02 ± 0.00	−7.19	<0.0001	−0.00 ± 0.00	0.30	0.77
Body mass	2.47 ± 1.21	2.04	0.041	0.59 ± 1.91	0.31	0.76	3.46 ± 1.85	1.87	0.061	−0.69 ± 1.27	−0.54	0.59
Air Temp	0.69 ± 0.88	0.79	0.43	−1.51 ± 1.65	−0.92	0.36	4.29 ± 1.11	3.88	0.0001	0.84 ± 1.04	0.80	0.42
Treatment x Phase	−0.14 ± 0.29	−0.48	0.63	0.30 ± 0.50	0.61	0.54	1.31 ± 0.35	3.70	0.0002	−0.05 ± 0.29	−0.15	0.88

b. Long-term impacts												
Intercept	5.12 ± 4.67	1.10	0.27	−20.13 ± 8.52	−2.36	0.018	1.55 ± 5.71	0.27	0.79	−21.82 ± 4.95	−4.41	<0.0001
Treatment – Control	−0.14 ± 0.16	−0.88	0.38	−0.63 ± 0.26	−2.43	0.015	−0.387 ± 0.25	−1.53	0.13	−0.28 ± 0.13	−2.09	0.037
Days of Manipulation	−0.01 ± 0.01	−1.78	0.075	−0.02 ± 0.01	−1.42	0.16	−0.01 ± 0.01	−0.92	0.40	0.01 ± 0.01	1.25	0.21
Clock Time (min from midnight)	0.00 ± 0.00	0.24	0.81	−0.00 ± 0.00	−0.25	0.80	−0.00 ± 0.00	−3.13	0.0018	−0.00 ± 0.00	−1.25	0.21
Body mass	−1.81 ± 0.77	−2.35	0.019	0.70 ± 1.22	0.57	0.57	−3.16 ± 1.02	−3.09	0.002	0.83 ± 0.65	1.28	0.20
Air Temp	−0.08 ± 0.25	−0.32	0.75	0.94 ± 0.46	2.05	0.040	0.25 ± 0.30	0.82	0.41	1.13 ± 0.27	4.24	<0.0001
Day type – post-handling	−0.07 ± 0.09	−0.73	0.46	−0.33 ± 0.16	−2.12	0.034	0.01 ± 0.14	0.07	0.95	−0.20 ± 0.20	−2.08	0.038
Hold – Weighed only	0.10 ± 0.16	0.59	0.55	0.02 ± 0.26	0.09	0.93	0.017 ± 0.25	0.70	0.48	−0.25 ± 0.13	−1.87	0.061
Treatment × Days of Manipulation	–	**0.23**	**0.64**	–	**0.08**	**0.77**	0.05 ± 0.01	3.67	0.0002	–	**0.11**	**0.75**
Day type × Hold	–	**2.34**	**0.13**	–	**1.61**	**0.20**	−0.42 ± 0.19	−2.26	0.024	–	**0.42**	**0.52**
Day type × Treatment	–	**0.15**	**0.70**	–	**0.07**	**0.80**	-	**0.27**	**0.10**	–	**2.24**	**0.13**

### 3.3 Long term impacts of enrichment withdrawal on surface temperature

In models of Eye and Face temperature, there were significant main effects of Days of Manipulation, indicating temperature increase through the experimental phase, but no interaction or main effect of Treatment (Table 3, Fig 2). In the Comb model, temperature decreased in control hens, but was unchanged in the barren hens, with a 1.3 °C difference evident by the trial end (Table 3, Fig. 2).

**Table 3:** Long-term effects (over 2–22 days) of enrichment withdrawal on 2682 comb temperature, 2682 face temperature and 2644 eye temperature measurements from 56 hens. Key explanatory variables are underlined; other variables control for behaviour and body position relative to the camera at measurement. Data were analysed using LMM with hen identity as a random effect. Test statistics in bold refer to interactions removed from the model based on a non-significant Likelihood Ratio Chi-squared test.

Fixed effects	Comb			Face			Eye		
	Coef ± S.E.	*T*	*P*	Coef ± S.E.	*t*	*P*	Coef ± S.E.	*t*	*P*
Intercept	6.81 ± 7.35	0.93	0.35	35.68 ± 1.66	21.52	<0.0001	26.30 ± 1.78	14.76	<0.0001
Treatment – Control	0.24 ± 0.51	0.48	0.64	0.05 ± 0.08	0.61	0.54	−0.09 ± 0.07	−1.32	0.19
Days of Manipulation	−0.03 ± 0.01	−2.34	0.019	0.01 ± 0.00	4.90	<0.0001	0.02 ± 0.00	5.22	<0.0001
Distance to camera	−0.59 ± 0.06	−10.12	<0.0001	−0.51 ± 0.01	−38.02	<0.0001	−0.22 ± 0.02	−14.92	<0.0001
Active – Yes	−0.42 ± 0.16	−2.70	0.0069	−0.20 ± 0.04	−5.58	<0.0001	−0.30 ± 0.04	−7.67	<0.0001
Cage pecking – Yes	0.19 ± 0.24	0.80	0.42	0.07 ± 0.06	1.21	0.23	0.22 ± 0.06	3.74	0.0002
Food-oriented – Hoppers	−0.05 ± 0.22	−0.24	0.81	0.18 ± 0.05	3.55	0.0004	0.82 ± 0.06	14.95	<0.0001
Food-oriented – Litter	−0.70 ± 0.21	−3.24	0.0012	−0.12 ± 0.05	−2.48	0.013	0.32 ± 0.05	6.01	<0.0001
Looking – Yes	−0.34 ± 0.13	−2.56	0.011	−0.08 ± 0.03	−2.44	0.015	−0.03 ± 0.03	−0.84	0.40
Head angle – Down	0.14 ± 0.19	0.72	0.47	−0.05 ± 0.04	−1.19	0.23	0.13 ± 0.05	2.75	0.006
Head position – Up	−0.20 ± 0.25	−0.79	0.43	−0.12 ± 0.06	−2.12	0.034	−0.12 ± 0.06	−1.94	0.053
Head tilt - Side on	−0.42 ± 0.20	−2.12	0.034	0.46 ± 0.05	10.14	<0.0001	−0.01 ± 0.05	−0.13	0.89
Head tilt – Away	−1.43 ± 0.38	−3.78	0.0002	0.48 ± 0.09	5.45	<0.0001	−0.10 ± 0.10	−1.04	0.30
Side – Right	−0.18 ± 0.12	−1.47	0.14	−0.01 ± 0.03	−0.18	0.86	0.00 ± 0.03	0.03	0.97
Clock Time (min from midnight)	0.00 ± 0.00	0.47	0.64	0.00 ± 0.00	6.58	<0.0001	0.00 ± 0.00	3.72	0.0002
Body mass (g)	3.10 ± 1.72	1.81	0.071	0.37 ± 0.33	1.11	0.27	0.71 ± 0.32	2.26	0.024
Air Temperature (°C)	1.33 ± 0.38	3.52	0.0004	0.02 ± 0.09	0.17	0.87	0.18 ± 0.10	1.85	0.064
Comb size	0.00 ± 0.00	0.34	0.74	0.00 ± 0.00	0.48	0.64	−0.00 ± 0.00	−0.72	0.47
Day type – Post-handling	0.07 ± 0.18	0.39	0.70	0.18 ± 0.03	5.87	<0.0001	0.13 ± 0.03	3.81	0.0001
Hold – Weighed only	−0.24 ± 0.50	−0.48	0.64	0.08 ± 0.08	0.99	0.33	−0.03 ± 0.07	−0.35	0.72
Treatment × Days of Manipulation	−0.07 ± 0.02	−4.12	<0.0001	-	**1.27**	**0.26**	-	**1.12**	**0.15**
Day type × Hold	0.72 ± 0.23	3.14	0.0017	-	**0.72**	**0.39**	-	**0.32**	**0.58**
Day type × Treatment	-	**0.25**	**0.62**	-	**0.02**	**0.88**	-	**0.01**	**0.91**
Treatment × Hold	-	**3.23**	**0.07**	-	**1.77**	**0.18**	-	**0.11**	**0.74**

**Fig. 3:**
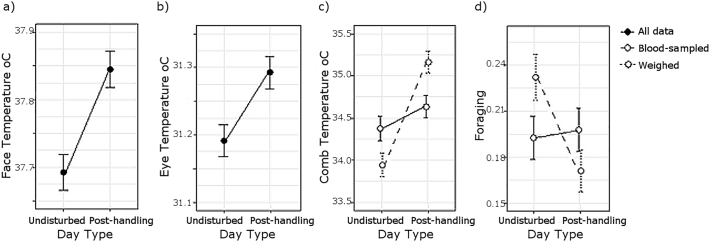
Differences between undisturbed and post-handling days in a) Face temperature, b) Eye temperature, c) Comb temperature and d) observations of foraging behaviour. In c) and d) data are subdivided into blood sampled versus weighed handling categories. Points indicate the mean ± 1 S.E.

With regards the impacts of intermittent handling, significant main effects Day Type indicate that Eye and Face temperature were higher on post-handling than undisturbed days (Table 3, Fig 3). In the Comb model only, there was a significant interaction of Day type x Hold: blood sampled birds had warmer combs on undisturbed days, but weighed birds showed a greater increase in comb temperature on post-handling days (Table 3, Fig 3c). Interactions were otherwise non-significant.

Larger hens had higher Eye temperature, otherwise Body Mass and Comb Size were non-significant covariates in these models (Table 3). Small fluctuations in Air Temperature during the trial (18.3 ± 0.4 °C) were positively correlated with Comb temperature (Table 3). The main effect of Clock Time was non-significant (Table 3). Covariates included to control for experimental noise are summarised in Table 3.

### 3.4 Long term impacts of enrichment withdrawal on behaviour

**Fig. 4:**
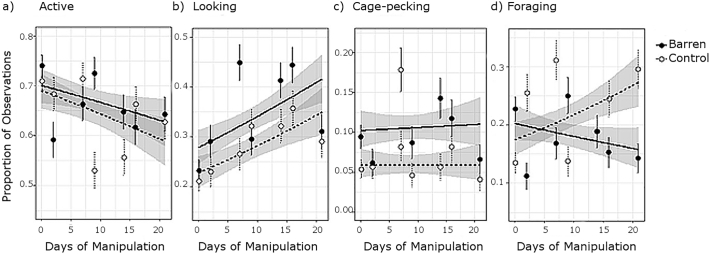
Logistic regression curves of the proportion of observations in which hens were classified into each of four binary behavioural categories, over days from enrichment withdrawal: a) Active, b) Looking, c) Cage-Pecking and d) Foraging. Data at 0 on the x axis corresponds to baseline data. On the y axis, 1 indicates an observation of the behaviour with 0 for any other behaviour. Due to the large number of data points, the mean ± 1 S.E. per sampling day are instead shown for clarity. Shading represents the confidence interval.

Activity was lower in heavier hens, but did not vary significantly with Treatment, Days of Manipulation or the interaction of these variables (Table 2b, Fig 4a). However, the focus of activity differed between treatments: barren hens showed higher rates of cage pecking and looking into the neighbouring pen, and a decrease in foraging over Days of Manipulation (Table 2b, Fig 4b–d). Foraging also decreased within days, and was lower in heavier birds (Table 2b). The main effects of Clock Time, Body Mass and Days of Manipulation were otherwise non-significant (Table 2b).

With regards the impact of intermittent handling, foraging mirrored long term patterns observed in comb temperature: a significant Day Type x Hold interaction indicates that blood sampled hens foraged less than weighed hens on undisturbed days, but that the foraging rate of weighed hens lowered on post-handling days (Table 2b, Fig 3d). In contrast, looking and cage pecking were significantly lower on post-handling than undisturbed days but did not vary between Holds (Table 2b). The Treatment x Day type interaction was never significant; thus withdrawal of environmental enrichments did not lessen the impact of intermittent handling.

### 3.5 Long term effects of enrichment withdrawal on baseline corticosterone

**Fig. 5:**
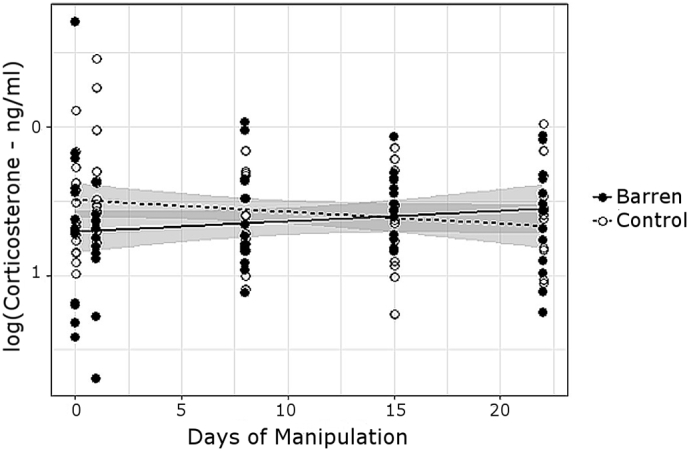
Change through the experimental phase in logged baseline corticosterone (ng/ml) within treatments. Confidence intervals are shaded around regression lines. Data at 0 on the x axis corresponds to baseline data.

Hens were blood sampled within 3 min of entry to the home pen and corticosterone values were independent of sampling latency within this period (Table 4), thus represent baseline values. Clock time, a measure also of number of hens hence disturbance experienced prior to sampling, was a non-significant covariate. Baseline corticosterone levels showed a Treatment x Days of Manipulation interaction, increasing over days in hens from barren pens but decreasing over days in hens from control pens (Table 4, Fig. 5). Modelled separately, however, the individual Treatment slopes were weak, and not significantly different to 0 (Barren: t_49_ = 1.40, p = 0.17, Control: t_50_ = 1.22, p = 0.23).

**Table 4:** Correlates of log-transformed baseline corticosterone data. Data from 135 corticosterone measurements from 28 individuals, analysed using a LMM with hen identity as a random effect.

Fixed effects	Log(Corticosterone - ng/ml)		
	Coef. ± S.E.	*T*	*P*
Intercept	0.055 ± 0.77	0.07	0.94
Treatment – Control	0.224 ± 0.10	2.23	0.035
Days of Manipulation	0.008 ± 0.00	1.57	0.12
Clock Time (min from midnight)	0.001 ± 0.001	0.92	0.36
Body Mass	−0.116 ± 0.39	−0.30	0.77
Latency to sample	0.000 ± 0.00	0.32	0.75
Days of Manipulation × Treatment	−0.015 ± 0.01	−2.21	0.029

### 3.6 Other analyses

Hens grew during the trial, gaining (mean ± s.d.) 0.08 ± 0.05 kg from baseline and also gaining weight within days, but neither body mass nor growth rate differed between Treatments (LME Body Mass ~ Treatment x Days of manipulation + Clock Time; dropped interaction: χ2 = 1.08, p = 0.30; Treatment: t = 1.15, p = 0.26; Days of Manipulation: t = 15.35, p < 0.0001; Clock Time: t = 2.24, p = 0.026).

## 4 Discussion

Withdrawal of enrichments and intermittent handling were associated with long-term increases in comb, face and eye surface temperature. Initially, we observed a drop in comb, face and eye temperature at 2–2.5 h of the enrichment withdrawal, suggesting acute stress [14]. In the longer term, behavioural changes that may indicate frustration [30,38] became apparent in the barren pens. In addition, we found weak but significantly cross-over effects of Treatment x Days of Manipulation on baseline corticosterone levels, decreasing through time in control pens but increasing in barren pens. Together, these changes in established welfare markers support enrichment withdrawal as an effective welfare manipulation. Comb temperature declined through the experimental phase in control but not barren pens thus was elevated in barren-housed hens by the trial end. In both treatment groups, face, eye and, in hens subject to a mild (weighing) handling protocol, comb temperature were also elevated on the day following weekly handling. On “undisturbed” days, a full six days from the last handling event however, comb temperature was higher in hens subject to a more severe (blood sampling) handling protocol. Together these data support elevated comb, face and eye surface temperature as indicators of chronic and intermittent stress exposure.

The impacts of enrichment withdrawal on cage pecking and looking were evident throughout the experimental phase, but effects on foraging, comb temperature and baseline corticosterone levels emerged over weeks. These more gradual changes may indicate a cumulative impact of withdrawal on stress levels. Alternatively, these variables may have been maximal in younger birds, limiting the scope for differentiation with treatment until age-related declines had occurred in the control pens. Hens were recruited shortly after the onset of laying, when reproductively mature but still growing. In support of the latter explanation, previous studies have also observed declines in baseline corticosterone through this developmental period [52]. We would predict that fully mature hens would show age-independent differences in comb surface temperature.

Behavioural changes observed in the experimental phase largely validated the surface temperature data. In barren pens, hens showed higher levels of cage pecking, spent more time looking into neighbouring pens, and showed a decrease through the course of the experimental phase in foraging behaviour relative to control birds. De Jong and colleagues [10] interpret “object pecking” (of caging, feeders and enrichment objects) such as cage pecking as a sign of frustration. Foraging on the other hand is an established indicator of positive environmental stimulation in hens [30]. Consistent with this expectation, we observed increases in foraging with litter renewal (immediately following enrichment withdrawal) that were most pronounced in control pens that had access also to foraging enrichments. Thus cage pecking, looking into the neighbouring (enriched) pen and reduced foraging in barren-housed hens all suggest long term frustration induced by enrichment withdrawal. Interestingly, cage pecking and looking also correlated positively with slight variations in air temperature (18.3 ± 0.4 °C): if the observed increases in comb temperature with both treatment and air temperature reflect core heat dissipation, then these behaviours may be influenced by body temperature in general.

Corticosterone showed weak but directional changes that mirrored treatment patterns in comb temperature. Specifically, we observed an expected (but non-significant) decline with age in control hens [52], but a significantly opposite pattern in the barren environment hens. In contrast, studies comparing hens with and without perches, bathing/foraging substrate and/or nest boxes showed no long-term reduction in plasma or egg corticosterone (mature hens: [2]; pullets: [51,52]). Similarly, movement from free-range housing to cages had only short-lived [27] or no effects on plasma corticosterone [9]. One explanation may be that the enrichments in those studies were ‘out of sight, out of mind’, whereas visible but inaccessible enrichments, as here, induce longer term frustration [38], as supported by our behavioural observations. Alternatively, we may be capturing an egg laying effect. Cronin et al. [9] noted that chickens disturbed or unable to settle just prior to laying had temporarily elevated corticosterone levels. By blood sampling in the late morning, we may capture the tail end of acute responses to laying in the absence of nest boxes/material. One hen here (excluded from corticosterone analyses) and two from contemporaneous research laid immediately after blood sampling, and had corticosterone levels >2.9 s.d. above the mean (mean ± s.d.: 1.60 ± 0.66; egg layers: 4.42 ng/ml, 8.78 ng/ml, 8.91 ng/ml), where another hen study reports corticosterone levels above ~3 ng/ml for acute stress [17]. Whilst long term corticosterone supplementation can cause body mass and muscular changes in chickens [47] that may increase metabolic heat production, this is not the mechanism underlying our thermal results, as body mass had no effect on comb temperature, and neither body mass nor growth rate differed between treatment groups.

In our models of behaviour and surface temperature, we specified two interactions to test whether intermittent handling and enrichment withdrawal had a multiplicative impact on welfare. These were: Treatment x Day type (undisturbed versus post-handling) and, to look at the intensity of the handling experience, Treatment x Hold (weighed versus blood-sampled; interaction in surface temperature models only). These interactions could not be included in corticosterone analyses, where data was necessarily limited to handling days and blood-sampled hens, respectively. As such, we cannot test whether the long-term increases in baseline corticosterone in the barren pens were contingent on those hens also experiencing blood sampling. On the undisturbed days (6 days from last handling), blood sampled hens had higher comb temperature and lower foraging rates than weighed birds, suggesting higher stress levels. However, neither of the above interactions was ever significant. Therefore, Day type and Hold effects were similar within each treatment group, and consequently provisioning with enrichments did not mitigate the negative impacts of intermittent handling (in contrast to Nazar & Marin [34]). Whilst absolute stress levels may be higher in blood-sampled hens, we would predict that the direction and magnitude of treatment effects on corticosterone would be similar in weighed birds.

The drop in comb, face and eye surface temperature in the hours immediately following enrichment withdrawal lends further weight to studies advocating skin cooling as a real-time marker of acute stress [14,19,22,45]. The elevation in face and eye temperature on post-handling days suggests that there is also a window of opportunity to detect past acute stressors using skin warming. Previous studies find increases in core body temperature [5] and disturbance to circadian core temperature rhythms [25] 24 h after an acute stressor, but this is the first to identify a similar surface temperature effect using IRT. In addition, on undisturbed days, we observed higher comb temperature in hens subjected to the more invasive blood sampling than weighing protocol. As observed with comb cooling measured immediately following acute stressors therefore [19], the magnitude of longer term comb warming appears to be proportional to stressor intensity. Seemingly in conflict with these results, weighed hens showed greater changes in comb temperature and foraging rate between undisturbed and post-handling days than blood-sampled hens. However, this may be due to higher “baseline” (undisturbed) stress levels in blood-sampled hens, reducing the scope for increase. Alternatively, there may be chronic stress-induced down regulation or exhaustion of acute stress responsiveness in blood sampled birds [42]. As such, comb temperature in undisturbed hens may be useful not only to the identification but quantification of chronic stress.

As with acute stress [19], the strongest and most consistent effects were in the comb. The chicken comb has a higher density of arteriovenous anastomoses (AVAs) than the eye or face: large vessels that bypass capillary beds. The constriction and dilation of comb AVAs facilitate rapid changes in blood flow hence heat exchange between the core and periphery in chickens [49]. This thermoregulatory role is evident in the positive correlation (slope: 1.33 ± 0.38) between comb temperature and slight air temperature variation (18.3 ± 0.4 °C) during the trial. Before discussing its potential as a welfare indicator therefore, evidence of stress-induced comb warming and the thermoregulatory processes this represents has immediate commercial implications, where the energetic costs of heat loss from the comb are well known from the practice of comb trimming to improve feed conversion efficiency and egg production [1]. Crucially, further work is required to test our assumption that comb temperature elevation reflects core temperature elevation, to fully understand both the welfare and commercial costs of chronic stress.

Regarding comb temperature as a welfare indicator, there are already promising tools in development for the automation of behavioural monitoring via overhead cameras [44]. As thermographic equipment becomes increasingly affordable, substituting visual for thermal cameras could potentially combine this behavioural with thermal data, to tease apart stress-induced from thermoregulatory, environmental and infectious causes of temperature change. Thus for both practical and scientific reasons, the comb has the greatest utility for welfare monitoring amongst available bare skin regions. Key to application will be demonstrating that stress-induced changes exceed individual-level and environmentally-induced variation in body temperature [22,29]. Promisingly, we observed no effects of body mass or comb size on comb temperature: proxies of individual-level capacity for metabolic heat production or dissipation, respectively. With regards the environment, whilst environmental temperature feeds back into the control of surface blood flow, as core temperature passes biologically important thresholds for tissue function, its influence becomes greater than that of environmental temperature [18,50]. As such, wild animals subject to great environmental variability can still exhibit detectable surface temperature changes with acute stress-induced hyperthermia [12,22]. To apply IRT more broadly to acute stress measurement in wild animals, there is a need to determine the margins above which thermoregulatory demands take precedence for individuals of e.g. different size or insulation [29]. Similarly, to develop IRT as a technique for chronic stress monitoring in wild animals, the next step must be to understand the how surface temperature relates to core temperature, to identify the energetic and thermoregulatory thresholds above which core temperature is maintained by heat dissipation, hence correlated to surface warming, rather than conservation and hence surface cooling [23]. For indoor housed farm and laboratory species however, where animals have set diets and are maintained within a narrow thermal range, we anticipate that surface temperature increase may be a general marker of environmental stress or frustration, human disturbance, and other psychological stressors that have been documented to increase core temperature [4,26].
